# Invasibility of a Nutrient-Poor Pasture through Resident and Non-Resident Herbs Is Controlled by Litter, Gap Size and Propagule Pressure

**DOI:** 10.1371/journal.pone.0041887

**Published:** 2012-07-24

**Authors:** R. Lutz Eckstein, Diana Ruch, Annette Otte, Tobias W. Donath

**Affiliations:** Institute for Landscape Ecology and Resource Management, Research Centre for BioSystems, Land Use and Nutrition (IFZ), Justus Liebig University Giessen, Gießen, Germany; Duke University, United States of America

## Abstract

Since inference concerning the relative effects of propagule pressure, biotic interactions, site conditions and species traits on the invasibility of plant communities is limited, we carried out a field experiment to study the role of these factors for absolute and relative seedling emergence in three resident and three non-resident confamilial herb species on a nutrient-poor temperate pasture. We set up a factorial field experiment with two levels each of the factors *litter cover* (0 and 400 g m^−2^), *gap size* (0.01 and 0.1 m^2^) and *propagule pressure* (5 and 50 seeds) and documented soil temperature, soil water content and relative light availability. Recruitment was recorded in spring and autumn 2010 and in spring 2011 to cover initial seedling emergence, establishment after summer drought and final establishment after the first winter. Litter alleviated temperature and moisture conditions and had positive effects on proportional and absolute seedling emergence during all phases of recruitment. Large gaps presented competition-free space with high light availability but showed higher temperature amplitudes and lower soil moisture. Proportional and absolute seedling recruitment was significantly higher in large than in small gaps. In contrast, propagule pressure facilitated absolute seedling emergence but had no effects on proportional emergence or the chance for successful colonisation. Despite significantly higher initial seedling emergence of resident than non-resident species, seed mass and other species-specific traits may be better predictors for idiosyncratic variation in seedling establishment than status. Our data support the fluctuating resource hypothesis and demonstrate that the reserve effect of seeds may facilitate seedling emergence. The direct comparison of propagule pressure with other environmental factors showed that propagule pressure affects absolute seedling abundance, which may be crucial for species that depend on other individuals for sexual reproduction. However, propagule batch size did not significantly affect the chance for successful colonisation of disturbed plots.

## Introduction

The susceptibility of a resident plant community to the establishment of new species (i.e. community invasibility) depends on the suitability of the habitat, biotic interactions with residents and the amount of resources available to new species [1]–[4]. Local environmental conditions are affected by climate [5] and land use changes that may trigger the establishment of newly arriving species (e.g., [6], [7]). Disturbance events (*sensu* [8]) that lead to the destruction of plant tissues and the creation of gaps in an intact vegetation usually increase resource availability, either directly through leakage from damaged tissues or indirectly through reducing the amount of resources captured by residents [9]. Additionally, disturbance will create competition-free space which may also benefit the establishment of new species [10]. Consequently, seedling emergence and survival is significantly higher in gaps than in intact vegetation across a range of habitat types such as semi-arid grasslands, temperate grasslands, temperate and tropical forests ([11] and references therein; but see [12]).

The quality of gaps, i.e. the magnitude of changes in resources (release from resource competition) and conditions (light, temperature) in comparison with intact vegetation as well as gap longevity, depends on gap size [11]. Therefore, seedling emergence is often higher in large than in small gaps (e.g., [13]–[15]). Pronounced and long-lasting enrichment in resource supply will have the largest effects on invasibility (cf. fluctuating resource hypothesis, [9]). This prediction was supported by field experiments showing that high levels of disturbance that were coupled with high fertility were most favourable for the establishment success of non-resident species [10], [16]–[19]. However, the response of different species to gaps and disturbance is idiosyncratic and seems to be related to plant traits such as seed mass [2], [18], [20], [21], life history stage [4] and germination requirements [2], [18] at least initially [22].

In semi-natural grasslands, the lack of regular disturbance through mowing (i.e. abandonment) leads to the accumulation of dead plant remains (litter), which may hamper seedling establishment [23]. This is confirmed by experimental studies demonstrating an increase in seedling (re)-establishment and species richness after litter removal (e.g., [24]–[27]). However, there is an increasing body of evidence from pot and field experiments for positive litter effects on seedling emergence and survival under stressful environmental conditions such as drought [25], [28]–[32]. Consequently, a number of non-resident (invasive) plant species may establish in grassland communities after abandonment (e.g., [33]–[35]).

However, the success of these invasive, non-resident species is also closely related to propagule pressure [36], which can be divided into propagule batch size (the number of individuals in a batch of arriving propagules) and propagule batch number (the number of propagule batches arriving at a location per unit time). In a meta-analysis, propagule pressure was identified as a strong and consistent predictor of the success of invasions (in terms of invasibility and invasiveness) at two different stages of invasion (establishment/spread and abundance/impact) across a range of taxa [37]. For plants, seed density together with alterations of water availability facilitated the invasion of *Holcus lanatus* into California dry coastal grassland [38]. Similarly, propagule pressure had a much stronger effect on invasibility of temperate forests in Virginia than biotic and abiotic properties of the recipient ecosystem [39].

However, inference concerning the relative effects of propagule pressure, biotic interactions, site characteristics and species traits on invasibility of plant communities is still limited [36]. This is because most seed sowing experiments (usually concerned with seed limitation) have been on native species only [40] and studies on non-native or non-resident species usually used only one sowing density (e.g., [10], [41]). Additionally, the effects of litter (accumulating as a consequence of land use changes in dry and temperate grasslands, e.g., [26]) for invasibility has – to our knowledge – only been studied for two *Solidago* species in old-fields [20]. Therefore, in the present study we addressed the effects a factorial manipulation of gap size, propagule pressure and litter cover on three phases of seedling establishment of six confamilial grassland herbs, three resident and three non-resident in the studied pasture community, in a one-year field experiment.

We addressed the following questions:

1. Are there significant effects of gaps size, propagule pressure, litter cover and species identity and which of these factors are most important for (i) the proportion and (ii) the absolute number of emerging seedlings and (iii) the chance of successful establishment (i.e. the presence of at least one surviving individual per plot)?
2. Are there significant and consistent differences in the abundance of emerging seedlings between resident grassland and non-resident ruderal species in response to these factors?
3. Do the effects of the studied factors vary between the stages of establishment (spring seedling emergence, seedling establishment after summer, seedling establishment one year after germination)?

## Materials and Methods

### Permissions

No specific permits were required for the described field studies. The land owner, a local farmer, who has leased the field site to the first author was informed about the planned activities and has given his consent. After the completion of the experiment, non-resident plants were removed and the experimental site was again used as sheep pasture. The field studies did not involve endangered or protected species.

### Study Species

We chose six species of the Asteraceae, three of which are typical herbs of extensively managed, infertile and dry to moderately fertile mesic acidic meadows and pastures [42]: *Crepis capillaris*, *Hypochoeris radicata* and *Leontodon autumnalis* (in the remainder species will be designated by their generic name). These three species occurred on the study site (resident species, Table 1). Additionally, we used two native ruderal species (*Picris hieracioides* and *Senecio jacobaea*) and the non-native *Solidago canadensis*, which do not occur on the study site but can be found in the vicinity along road verges and in old-fields (non-resident species). Species differ with respect to achene mass (Table 1). We collected ripe achenes (for brevity called seeds in the remainder of the paper) of the species in field populations on or close to the study site. To cover a larger comparable seed pool of the study species, our seed collection was amended through seeds from a commercial seed supplier (Rieger-Hofmann GmbH, Blaufelden-Raboldshausen, Germany) to obtain material from two regions in central and southern Germany. All seeds were pooled for each species before seed batches (size: 5 and 50 seeds) were prepared. These were stored dry in Eppendorf tubes at room temperature until the start of the experiments.

**Table 1:** Status, achene mass, and establishment in the common garden.

Status	Species	Achene mass	se	Estab. control	se	Estab. litter	Se
Residents	*Crepis capillaris*	0.22	0.01	0.516	0.040	0.692	0.047
	*Hypochoeris radicata*	0.73	0.02	0.624	0.045	0.576	0.061
	*Leontodon autumnale*	0.74	0.02	0.584	0.032	0.608	0.068
Non-residents	*Picris hieracioides*	0.86	0.06	0.584	0.074	0.652	0.021
	*Senecio jacobaea*	0.26	0.01	0.200	0.028	0.196	0.055
	*Solidago canadensis*	0.05	0.003	0.212	0.063	0.220	0.054

Achene mass (n = 4–8), and proportion of established seedlings (Estab., after 161 days; n = 5) in pots in a common garden under control conditions (i.e. without litter cover) and with a litter cover of 400 g m^−2^ as in the field experiment. Analysis of variance showed that differences in establishment between species were significant (F_5,48_ = 27.0, p<0.0001), whereas litter effects and the species x litter interaction were not (p>0.23).

### Experimental Design

The field experiment was set up on a non-intensively used sheep pasture, located in a low-mountain region of Hessen (Germany; 50°45′57.48′′N, 8°40′28.45′′E) at 216 m above sea level. Mean annual precipitation in the region is about 700–800 mm and mean annual temperature ranges between 8 and 9°C [43]. The study site is a W-exposed gentle slope, characterised by a shallow layer of acidic loamy soil (pH_water_ = 4.89) over greywacke and slate. On a homogenous portion of the pasture we established 15 rows parallel to the slope with 20 plots per row. Square plots of 0.1 m^2^ (32×32 cm) and 0.01 m^2^ (10×10 cm) size (large and small gaps) were arranged in a checkerboard manner by removing all above ground vegetation. To avoid germination of species from the seed rain and transient seed bank, we additionally removed the upper 2 cm of soil in each plot. There was a buffer of undisturbed pasture of about 40 cm between plots; the distance between rows was 50 cm. The experimental substrate for the experiment that was used to refill the plots and make their surface level with the surrounding was the upper soil (pH_water_ = 3.57) from a nearby (20 m distance) forest edge, which was excavated and steam sterilised (6 h at 80°C; Sterilo 1 K; MAFAC/Schwarz, Alpirsbach, Germany) before use. We used a completely randomized experimental design to study the effects of *species identity* (factor levels [k] = 6; cf. Table 1), *litter cover* [k = 2; 0 (control) and 400 g grass litter m^−2^], *seed pressure* [k = 2; 5 and 50 seeds] and *gap size* [k = 2; 0.1 (large) and 0.01 m^2^ (small)] on seedling emergence. Grass litter was collected on a mesic unfertilised grassland site that contained none of the study species. Bench-dry grass litter was used; it was not oven-dried because this might change the chemical components. The applied amount of grass litter corresponds to the annual litter production of meadows with intermediate productivity [44]. In large plots, seeds were sown into the central 10×10 cm to keep seed density constant between gap sizes. For each species, the factorial combinations of *litter cover*, *seed pressure* and *gap size* was replicated six times, resulting in 48 plots and a total number of 288 plots. Seeds of all six species were sown on 7 December 2009 to allow for cold stratification potentially needed for germination. Additionally, twelve plots (three replicates of the factorial combination of gap size and litter cover) were left unsown but equipped with buried dataloggers (Tinytag Transit with internal sensor, Gemini Dataloggers Ltd, Chichester, UK) that measured hourly temperatures just below soil surface. During one occasion in early summer (18 June 2010), photosynthetically active radiation was measured on each sown plot above the litter layer (3 cm above soil surface) and above the pasture vegetation (ambient radiation) to compare relative light availability between large and small plots. One measurement was taken per plot using quantum sensors (Li-190, Licor Inc., USA). On two dates during the period of initial seedling growth (25 May and 23 June 2010), we took cylinder soil samples on large datalogger plots to a depth of 2 cm (n = 3). Soil was immediately transported to the laboratory in plastic bags, where fresh and dry (after 24 h at 105°C) mass were determined. These data were used to estimate gravimetric soil water content.

Three countings (factor *time*) of seedlings were carried out to cover different stages of the recruitment process: (i) initial seedling emergence in spring (21 April 2010), (ii) seedling establishment in autumn, i.e. after summer drought and a phase of seedling growth (12 October) and (iii) final establishment after one year (6 April 2011). Seedlings were marked with non-toxic colour and wooden sticks to be able to differentiate newly emerged seedlings from those already present. Beyond the absolute number of emerged seedlings, we also analyzed proportional emergence, which was estimated as the number of seedlings at each date divided by the number of germinable seeds. The latter was obtained by multiplying the number of seeds sown by the average proportion of seeds germinating under optimal conditions in a growth chamber experiment (night/day temperature of 10 and 20°C (12 h dark/12 h light), 5 replicate Petri dishes with 50 seeds on wetted filter paper per species, germination after 14 days).

We compared the quality and persistence of gaps of different size using the amount of regrowth into the experimental plots during the course of the experiment. To this end, plant biomass was harvested at the end of the experiment, dried and weighed on all plots where no seedlings of the sown species had established.

### Common Garden Experiment

To test seedling emergence under competition-free outdoor conditions without water limitation, a randomized pot experiment was set up in a common garden close to Giessen (50°32′ N, 8° 41.3′ E, 172 m a.s.l.) at the same time as the field experiment. We sowed 50 seeds of each species into ten replicate pots (10×10 cm, 1 L volume) filled with commercial potting soil (Fruhstorfer Erde, Type P, Industrie-Erdenwerke Archut GmbH, Lauterbach, Germany). Half of the pots were covered with 400 g m^−2^ of grass litter. All pots were exposed to open-air conditions with additional watering. Emerged seedlings were counted on 14 April, 2 May, 26 May and on 25 July 2010; at each date emerged seedlings were removed from the pots.

### Statistical Analysis

To account for possible non-independence between counting dates, a five-way fixed effect repeated measures ANOVA was employed to test for the effects of the four between-subjects factors *species identity*, *litter cover*, *seed pressure* and *gap size* and the within-subject factor *time* on the three stages of seedling emergence. In a first step, all three counting dates are analyzed together using a MANOVA approach. In a second step, univariate analyses can be done to test how significant factors affected seedling emergence in the three phases of the life cycle. Data were arcsine-transformed to improve normality and homogeneity of variances. One species (*Solidago*) had to be omitted from the analysis since establishment failed completely. Since non-resident species are considered to take advantage of large disturbances [9], i.e. large gaps, or abandonment (e.g., [33]), i.e. litter cover, we calculated a planned contrast between the groups of resident and non-resident species (i) across all other factors, (ii) for the treatment combination large plots plus litter cover and (iii) for the treatment combination large plots without litter cover.

Data on soil water content were analysed with a two-way GLM-ANOVA testing for the effects of *date* and *plot size*. For analysis of light measurement we used a three-way GLM-ANOVA testing for potential differences between plots assigned to different gap sizes, seed pressures and litter treatments. Biomass per unit plot area at the end of the experiment was analysed using a two-way GLM-ANOVA.

Since we were not only interested in potential effects of the studied factors on the abundance of seedlings but also on the simple chance of establishing at least one surviving individual in a plot after one year, we analysed contingency tables (counting the presence of at least one individual as success) using chi-square tests and estimated odds ratios and their 95% confidence limits to test for the independence of (i) *gap size* and *litter*, (ii) *gap size* and *seed pressure* and (iii) *litter* and *seed pressure* [45]. All statistical analyses were done using Statistica 10.0 [46].

## Results

### Field Experiment

#### Effects on abiotic conditions

**Figure 1:**
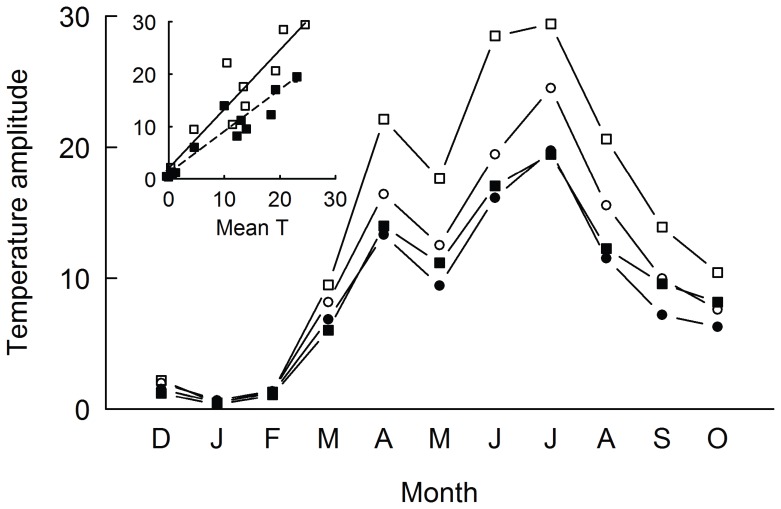
Average monthly temperature amplitude at soil surface. Temperature amplitude in large (0.1 m^2^, squares) and small plots (0.01 m^2^, circles) without (controls, open symbols) and with a litter cover of initially 400 g m^−2^ (filled symbols) throughout the first eleven months of the experiment. Inset figure shows the correlation between monthly temperature amplitude (y-axis) and monthly mean temperature (x-axis) of large plots with and without litter. The dashed and the continuous line show linear regressions for large plots with litter and large control plots, respectively; t-test of slopes: t = 2.183, df = 18, P = 0.0425.

**Figure 2:**
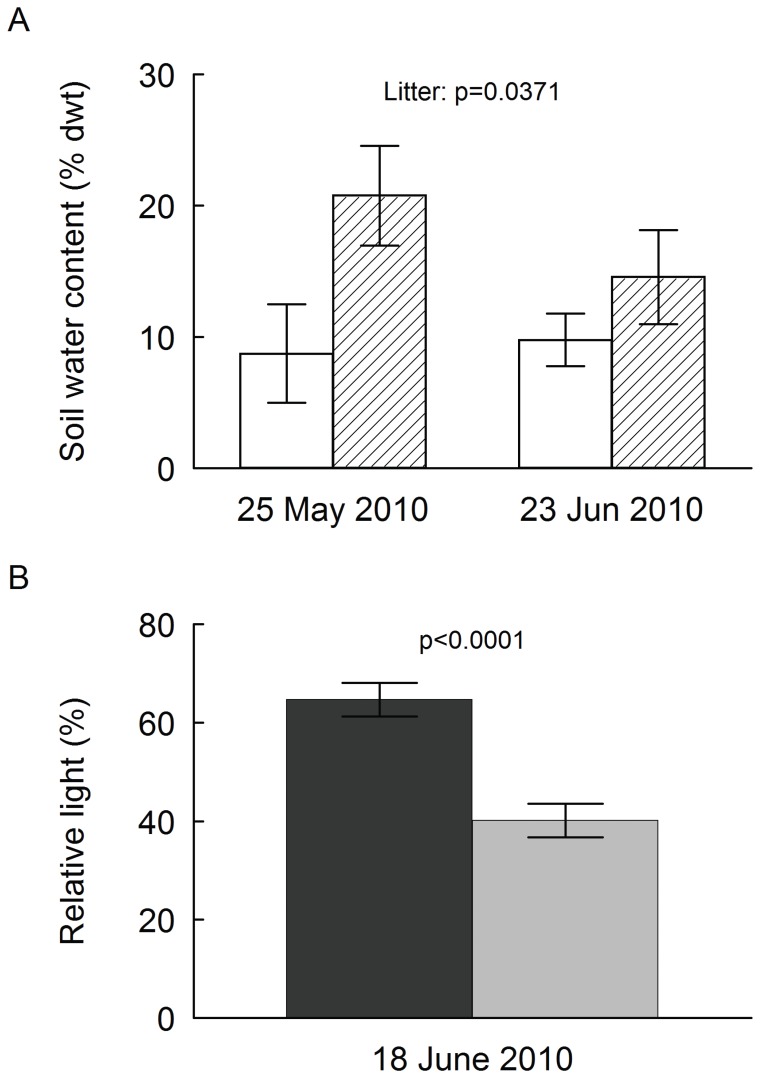
Soil water content and light availability. Gravimetric soil water content (A) in large plots (0.1 m^2^) at two occasions during the time of initial seedling growth (25 May, 23 June) and relative light intensity (B) in large and small plots (0.01 m^2^) ca. 3 cm above the soil surface on 18 June. In a., white bars denote control plots (without litter), hatched bars plots with litter (initially 400 g m^−2^). Data are means ± s.e., n = 3. Analysis of variance showed a significant litter effect (F_1,8_ = 6.24, p = 0.0371), whereas the effects of date and the litter x date interaction were not significant. In b., the black bar denotes large plots, the gray bar small plots. Data are means ± s.e., n = 150). Analysis of variance showed significant effects of plot size (F_1,288_ = 25.8, p<0.0001), whereas there were no effects of seed number sown, litter addition or any of the interactions.

Especially during the growing season, i.e. from May to September, soil temperature amplitude in the uppermost soil layer was consistently higher on control plots than in plots covered by litter (Fig. 1). Differences between gap sizes were small in plots with litter, whereas in control plots temperature amplitude was higher in large than in small gaps. Temperature amplitude increased with monthly mean temperature (Fig. 1, inset figure) and regression slopes differed significantly between large control plots and those with a litter cover (t-test of slopes: t = 2.183, df = 18, P = 0.0425). For soil water content in large plots, we found a significant effect of *litter cover* (F_1,8_ = 6.24, p = 0.0371; Fig. 2a), whereas the effects of *date* and the *litter* x *date* interaction were not significant. Relative light availability was significantly lower on small (40.2±3.4%, mean ± s.e., n = 150) than on large plots (64.7±3.4%; F_1,288_ = 25.8, p<0.0001; Fig. 2b).

### Proportional and Absolute Seedling Emergence

**Figure 3:**
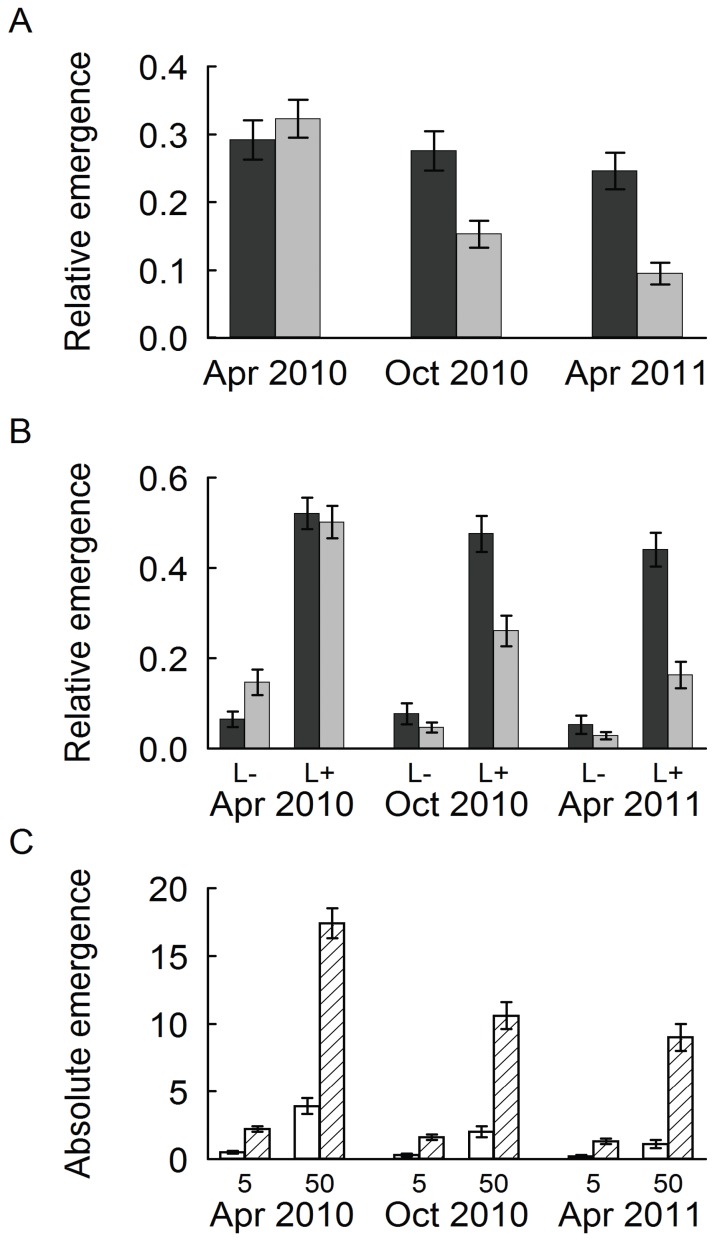
Average seedling emergence in relation to plot size, propagule pressure and litter. Seedling emergence (proportion of sown seeds) in large (0.1 m^2^, black bars) and small plots (0.01 m^2^, grey bars) (A), proportion of sown seeds differentiated according to the litter treatment (L-, controls; L+, 400 g litter m^−2^) (B), and absolute number of emerged seedlings in relation to propagule pressure (5 or 50 seeds sown) and litter cover (white bars, controls; hatched bars, 400 g litter m^−2^) (C) in spring 2010 (Apr 2010), after the first summer (Oct 2010) and one year after first emergence (Apr 2011). Data are means ± s.e., n = 120 in a. and 60 in b. and c.

There were consistent effects of *species*, *litter* and the *plot size* x *litter* interaction on proportional and absolute seedling emergence for all stages of recruitment (Table 2). Additionally, except for relative emergence in April 2010, relative and absolute seedling recruitment was significantly higher on large than on small plots (Fig. 3). Overall, *litter* was the most important factor, explaining always about 30% of the total variation. Litter effects on relative and absolute seedling emergence were generally positive (Fig. 3). In contrast, *species* accounted for 5–10% and the *plot size* x *litter* interaction for 1–6%. When considering absolute seedling emergence, more seedlings established on plots where 50 than on plots where 5 seeds had been sown (propagule pressure effect). For absolute emergence, MANOVA results revealed significant within-subject effects of *time*, *time* x *gap size*, *time* x *propagule pressure* and *time* x *species* x *litter*; for proportional emergence the effects of *time*, *time* x *species*, *time* x *gap size*, *time* x *litter*, *time* x *species* x *litter* and *time* x *gap size* x *litter* were significant (data not shown).

**Table 2:** Univariate results of a repeated measures ANOVA testing for the effects of species identity, plot size, seed number, litter and time on relative (A) and absolute (B) seedling emergence.

A) relative emergence		21 April 2010			12 October 2010			06 April 2011		
	Df	MS	P	Var. (%)	MS	P	Var. (%)	MS	P	Var. (%)
Intercept^MMM^	1	**60.48**	**<0.0001**		**31.07**	**<0.0001**		**21.15**	**<0.0001**	
Species(S)^MMM^	4	**1.27**	**<0.0001**	**10.6**	**0.46**	**0.0004**	**5.2**	**0.49**	**<0.0001**	**5.6**
Plot size (P)^MMM^	1	0.22	0.1190	0.5	**1.37**	**<0.0001**	**3.8**	**2.73**	**<0.0001**	**7.7**
Seed number (N)	1	0.05	0.4543	0.1	0.14	0.2108	0.4	0.23	0.0876	0.6
Litter (L)^MMM^	1	**20.51**	**<0.0001**	**42.7**	**12.20**	**<0.0001**	**33.9**	**10.75**	**<0.0001**	**30.2**
S*P	4	0.08	0.4855	0.6	0.03	0.8497	0.3	0.12	0.1904	1.3
S*N	4	0.10	0.3657	0.8	0.05	0.7009	0.5	0.03	0.8286	0.3
P*N^(M)^	1	0.01	0.7821	0.0	0.23	0.1070	0.6	**0.40**	**0.0235**	**1.1**
S*L^(M)^	4	**0.36**	**0.0035**	**3.0**	0.09	0.4099	1.0	0.07	0.4518	0.8
P*L^MMM^	1	**0.55**	**0.0141**	**1.1**	**0.74**	**0.0039**	**2.0**	**2.19**	**<0.0001**	**6.2**
N*L^M^	1	**0.39**	**0.0392**	**0.8**	**0.41**	**0.0308**	**1.1**	0.25	0.0707	0.7
S*P*N	4	0.05	0.7073	0.4	0.01	0.9727	0.1	0.008	0.9811	0.1
S*P*L	4	0.06	0.6135	0.5	0.10	0.3652	1.0	0.091	0.3192	1.0
S*N*L	4	0.02	0.9181	0.2	0.13	0.2144	1.4	0.064	0.5063	0.7
P*N*L	1	0.001	0.9099	0.0	0.04	0.5066	0.1	0.001	0.8962	0.0
S*P*N*L	4	0.15	0.157	1.3	0.04	0.7837	0.4	0.051	0.6169	0.6
Error	200	0.089		37.4	0.086		48.0	0.077		43.1
Total	239									
**B) absolute emergence**		**21 April 2010**			**12 October 2010**			**06 April 2011**		
	**Df**	**MS**	**P**	**expV(%)**	**MS**	**P**	**expV(%)**	**MS**	**P**	**expV(%)**
Intercept^MMM^	1	**261.14**	**<0.0001**		**163.19**	**<0.0001**		**108.83**	**<0.0001**	
Species(S)^MMM^	4	**4.39**	**<0.0001**	**10.9**	**3.14**	**<0.0001**	**8.9**	**2.65**	**<0.0001**	**7.7**
Plot size (P)^M^	1	**1.98**	**0.0020**	**1.2**	**2.47**	**0.0022**	**1.7**	**4.70**	**<0.0001**	**3.4**
Seed number (N)^MMM^	1	**34.40**	**<0.0001**	**21.4**	**21.17**	**<0.0001**	**14.9**	**12.68**	**<0.0001**	**9.3**
Litter (L)^MMM^	1	**52.60**	**<0.0001**	**32.7**	**40.89**	**<0.0001**	**28.8**	**43.45**	**<0.0001**	**31.7**
S*P	4	0.23	0.3479	0.6	0.22	0.4863	0.6	0.31	0.2336	0.9
S*N	4	0.11	0.7143	0.3	0.46	0.1333	1.3	0.35	0.1787	1.0
P*N	1	0.72	0.0602	0.4	0.26	0.3162	0.2	0.22	0.3226	0.2
S*L^M^	4	**0.84**	**0.0030**	**2.1**	0.30	0.3234	0.9	**0.65**	**0.0224**	**1.9**
P*L^MMM^	1	**2.57**	**0.0004**	**1.6**	**1.83**	**0.0083**	**1.3**	**5.77**	**<0.0001**	**4.2**
N*L^MMM^	1	**1.27**	**0.0128**	**0.8**	**1.15**	**0.0357**	**0.8**	**2.99**	**0.0003**	**2.2**
S*P*N	4	0.13	0.6325	0.3	0.05	0.9432	0.1	0.10	0.7603	0.3
S*P*L^M^	4	0.16	0.5420	0.4	**0.75**	**0.0228**	**2.1**	**0.70**	**0.0153**	**2.0**
S*N*L	4	0.19	0.4241	0.5	0.37	0.2290	1.0	0.27	0.3145	0.8
P*N*L^(M)^	1	0.72	0.0595	0.4	0.34	0.2513	0.2	0.56	0.1138	0.4
S*P*N*L^M^	4	**0.56**	**0.0273**	**1.4**	0.30	0.3280	0.8	**0.57**	**0.0396**	**1.7**
Error	199	0.20		24.9	0.26		36.2	0.22		32.3
Total	238									

The significance of factors in an initial MANOVA (repeated measures approach), analyzing all three counting dates together is given in the first column: MMM = MANOVA p<0.001; MM = MANOVA p<0.01; M = MANOVA p<0.05; (M) = MANOVA p<0.1. Seedlings were counted in spring 2010 (21 April 2010), after the first summer (12 October 2010) and one year after the start of the experiment (08 April 2011). Data on relative and absolute emergence were arcsine- and fourth-root-transformed, respectively, before analysis; one species (*Solidago*) was removed because establishment failed completely. Abbreviations: df = degrees of freedom, MS = mean square, p = error probability, Var. (%) = percentage of explained variation. Significant effects are given in bold.

**Figure 4:**
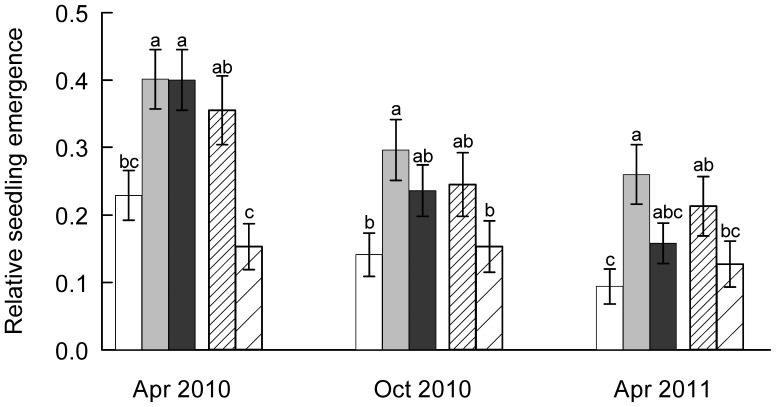
Average seedling emergence per species. Seed ling emergence (proportion of sown seeds) of the resident species *Crepis capillaris* (white), *Hypochoeris radicata* (gray), *Leontodon autumnale* (black) and the non-residents *Picris hieracioides* (densely hatched) and *Senecio jacobaea* (widely hatched) in spring 2010 (Apr 2010), after the first summer (Oct 2010) and one year after first emergence (Apr 2011). Data are means ± s.e., n = 48. Analysis of variance, calculated for each date separately, showed significant species effects (F_4,200_>5, p<0.0004). For each data, different letter denote means that are significantly different (at p<0.05) according to Tukey test.

With respect to species differences (Fig. 4), the contrast analysis showed significantly higher proportional seedling emergence of resident than non-resident species in April 2010 (F_1,200_ = 13.6, p<0.0001) but not for the other dates. When only considering large plots with and without litter cover, proportional seedling emergence was significantly higher in resident than non-resident species (April 2010: F_1,200_ = 5.06, p = 0.0256 and F_1,200_ = 5.14, p = 0.0245 for plots with and without litter cover, respectively; October 2010: F_1,200_ = 7.03, p = 0.009 for large control plots). Relative and absolute seedling establishment decreased with the stage of recruitment (Figs 3, 4; MANOVA significant *time* effects). *Hypochoeris*, *Leontodon* and *Picris* with a seed mass >0.7 mg showed consistently higher seedling emergence than *Crepis* and *Senecio* with a seed mass <0.3 mg, whereas *Solidago* (the species with the lowest seed mass) failed completely.

Additionally, there were factors that affected only certain stages of seedling recruitment (Table 2). The effects of different gap sizes were not significant for seedling emergence in April 2010, whereas significantly more seedlings were found on large plots in October 2010 and April 2011 (Fig. 3a; MANOVA significant *time* x *gap size* effects for absolute and proportional emergence). Figure 3b shows that significantly more seedlings were found on plots with litter cover but that for the later two stages of seedling recruitment, the effects of gap size were much larger on plots with litter cover than on controls.

The chi-square tests for lack of independence between factors were only significant for the species – litter comparison (chi-square = 12.8, df = 4, p = 0.0125) but not for any other factor combinations (data not shown). Similarly, although there were differences in the chance of successful establishment on large plots without and with litter, on small plots with 5 and 50 seeds and on plots without and with litter, the confidence range included the value of 1 and thus the odds between the groups compared was not significant at p<0.05 (Table 3).

**Table 3:** Odds ratio and 95% confidence range based on 2×2 contingency tables for three processes concerning the successful establishment of species.

Process	Odds ratio	Confidence range (95%)
Establishment on large plots without litter (controls) in comparison with litter	0.567	0.225–1.426
Establishment on small plots with 5 seeds in comparison with 50 seeds	0.640	0.294–1.391
Establishment on plots without litter with 5 seeds in comparison with 50 seeds	0.561	0.210–1.497

Successful establishment was defined as the presence of at least one individual on a plot in spring 2011. An odds ratio <1 indicates smaller odds for the first group compared to the second group. Since the confidence range includes the value of 1, the odds between the groups compared is not significant at p<0.05.

Biomass per square meter at the end of the experiment was significantly higher on small plots (111±6 mg m^−2^, n = 97) than on large plots (6±8 mg m^−2^, n = 77; F_1,170_ = 504, P<0.0001; data not shown).

### Common Garden Experiment

Analysis of variance showed that there were differences in establishment between *species* (F_5,48_ = 27.0, p<0.0001) with a higher proportion of seedling establishment in the resident (0.60±0.02, mean ± s.e., n = 30) than in the non-resident species (0.34±0.04; contrast F_1,48_ = 63.0, p<0.0001). However, there were no significant effects of *litter* and the *species* x *litter* interaction (p>0.23).

## Discussion

### Effects of Experimental Treatments on Abiotic Conditions and Competition

Large gaps persisted as low-competition patches throughout the first year after creation, whereas species from the resident vegetation surrounding the experimental plots re-colonised small gaps by means of vegetative growth (own observation). This is in line with [14] who found that small gaps were colonised more rapidly and showed a higher density of clonal ramets than large gaps. Owing to the removal of vegetation, the central 10×10 cm of the large gaps in our experiment were characterised by higher relative light availability during the period of seedling growth than small gaps. However, higher energy input into large gaps resulted in higher diurnal near-surface temperature amplitudes, which means that seedlings were subject to colder night and warmer day temperatures. High daytime temperatures resulted in lower soil water contents especially during summer as long as the soil was not protected from direct radiation through a litter cover. Thus, gap size and the presence of litter exert contrasting effects on biotic and abiotic conditions, which depending on the ambient environment may facilitate or impede seedling establishment and growth [11], [12], [15], [20], [23], [28].

### Effects of Experimental Treatments on Seedling Emergence

In the studied pasture with a shallow soil layer, *litter cover* was the factor with the largest effects on proportional and absolute seedling emergence (Table 2). Accumulation of litter may negatively affect germination and seedling establishment of plants [23]. For example, seedling establishment of two species of *Solidago* in an abandoned field was strongly inhibited through litter [20]. In contrast, in the present experiment, except for *Solidago*, litter cover presented shelter against high radiation and high daytime temperatures and thus facilitated seedling emergence of all other species. Our field data lend further support to results of controlled pot experiments that already demonstrated positive effects of litter under conditions of water limitation [28], [29], [31], [47], [48]. Similarly, the lack of a significant litter effect on seedling emergence in our common garden experiment, where pots were watered regularly to field capacity, presents further evidence that the sign of the litter effect strongly depends on abiotic conditions. There is evidence from a meta-analysis of litter effects on grassland vegetation that facilitation of seedling emergence was predominantly found in pot and some field experiments (i.e. situations with water limitation) whereas greenhouse experiments mostly reported negative litter effects (Loydi et al., unpublished data). However, above a threshold with respect to mass or thickness, litter accumulation will impede seedling emergence [28], [30], [49] and reduce species richness in semi-natural grasslands [24]–[26].

Propagule pressure (in this case propagule batch size, i.e. the number of sown seeds) had consistent and significant effects on the absolute number of emerged seedlings (Table 2). Similarly, the number of seedlings of *Holcus lanatus* in California coastal grassland increased significantly with propagule batch size [38]. Our levels of propagule pressure (500 and 5000 seeds per square meter) correspond roughly to the treatment levels in [38] of 555 and 4033 seeds added per square meter which differed significantly with respect to seedling emergence and survival. However, we did not find any significant effects of propagule pressure on the proportion of emerged seedlings. Additionally, although the chance for successful establishment in small plots or plots with litter was smaller for 5 than for 50 seeds, the odds ratio did not vary significantly between levels of propagule pressure (Table 3). Therefore, propagule pressure affected abundance of seedlings but not the chance of successful establishment. This can also be deduced from the figures in [38] which depict that also the lowest seed density treatment resulted in average establishment that differed from zero. High abundance of a new species in a community will probably increase its chance for successful establishment in the long run since highly abundant species may monopolise resources. Additionally, high numbers of individuals will benefit the long-term establishment of dioecious, outcrossing or self-incompatible species, which depend on another individual for sexual reproduction, whereas this is less important for selfing species.

For plants, propagule pressure and disturbance have been identified as important factors increasing the invasibility of communities [37]. However, except for the studies of [39] (manipulating propagule batch number) and [38] (manipulating propagule batch size), our study is the first attempt to empirically compare the effects of propagule pressure and other environmental factors relevant for species establishment. Taken together, relative and absolute seedling abundance was strongly facilitated through a moderate litter cover and large plot size (strong disturbance). Additionally, propagule pressure triggered absolute seedling abundance but not relative seedling abundance or the chance of establishment.

### Differences in Emergence between Resident and Non-resident Species

We found consistent and significant effects of species identity on seedling emergence (e.g., [28], [30], [49]), which may be due to status (non resident ruderals vs. resident grassland herbs) or species traits (e.g. seed size). Although planned contrasts showed significant differences in relative seedling emergence between resident and non-resident species, the status of species should not be overrated. Firstly, significant differences (with one exception) were only found for initial seedling emergence but disappeared in later stages of recruitment. Secondly, species specific effects were highly significant and consistent, whereas variation among species within each status group was large. For example, the non-resident *Picris* showed consistently higher relative seedling emergence than the resident *Crepis*. Therefore, differences in traits among species are probably better predictors for the species’ responses to experimental manipulations than status. Although we cannot draw definite conclusions based on six study species, our results suggest that small-seeded species may perform better under favourable site conditions in the field experiment (seedling emergence varied by a factor of 12 between (favourable) litter and (unfavourable) control plots in small-seeded species and by a factor of 6.5 in large-seeded species). The species with the smallest seed size (*Solidago*) failed completely despite successful seedling establishment in the common garden, i.e. under favourable conditions. Thus, a combination of environmental filters (stressful biotic and abiotic conditions) and species traits probably resulted in lack of seedling establishment in *Solidago*. It is unlikely that the habitat conditions were completely outside the species niche (e.g. considering the low soil pH) since *Picris* and *Senecio* established successfully despite an ecological optimum on neutral to basic soils [50]. In contrast, large-seeded may cope better with unfavourable conditions; two of the three large seeded species showed emergence of >15% in control plots. However, since there is still large variation within species groups according to status or seed size, additional factors may be responsible for the observed consistent species-specific response to environmental manipulations.Significant relationships between seed size and colonisation success were e.g. found in a multi-species field experiment in limestone grassland [18] and in synthesised grassland communities [2]. Similarly, among six monocarpic perennials, seedling emergence in trays with competition (by *Poa pratensis*) was higher in large-seeded (>1 mg) than in small-seeded species [21], whereas there were no differences in response to litter (1.2 cm of straw). However, results of a meta-analysis of litter effects shows significantly higher emergence of large- than small-seeded species in response to litter (Loydi et al., unpublished data). Better performance of large-seeded species under unfavourable conditions [51], [52] may be related to the fact that larger seeds give rise to larger seedlings (seedling size effect) and that larger seeds contain resources that may support seedlings during periods of carbon deficits (reserve effect, cf. [53]). An implication of the latter [53] is that the benefits of seed reserves are only temporary (at some point seed reserves are completely exhausted). Consequently, the reserve effect may induce a fitness advantage in relation to moderate amounts of litter since the hazard induced through a litter cover (e.g. shading) will decrease when litter decomposes e.g. [31] or with seedling length growth.

### Differences between Stages of Establishment

The effects of environmental factors varied among the three stages of establishment (spring seedling emergence, seedling establishment after summer, seedling establishment one year after germination). Whereas initial seedling emergence (Apr 2010) was facilitated through litter independent of gap size, the most suitable conditions for later stages of seedling emergence (Oct 2010, Apr 2011) were found on large plots with litter cover. This is because different conditions may be conducive for seed germination, seedling growth, survival and reproduction (e.g., [4], [27], [54]). Similarly, in a long-term grassland experiment, seed mass and germination traits were the best predictors of establishment success among 54 plant species after 2 years, whereas after 5 years these traits were unrelated to success of the invaders [22]. Also our results suggest (in agreement with the reserve effect hypothesis [53], cf. above) that initial seedling establishment from beneath a moderate litter cover, which in turn protects seeds and seedlings from desiccation, is related to seed mass. In later stages, i.e. after summer drought and a period of seedling growth, differences in emergence between large and small-seeded species decrease. At this stage, gap size, which is related to the area of competition-free space for seedling growth, becomes more important.
